# Tumor-derived VEGF modulates hematopoiesis

**DOI:** 10.1186/2040-2384-1-9

**Published:** 2009-12-23

**Authors:** Yuan Xue, Fang Chen, Danfang Zhang, Sharon Lim, Yihai Cao

**Affiliations:** 1 Department of Microbiology, Tumor and Cell Biology, Karolinska Institute 171 77 Stockholm, Sweden; 2 Shanghai Chest Hospital Affiliated to Shanghai Jiaotong University, 200030 Shanghai, PR China

## Abstract

VEGF-induced angiogenesis significantly contributes to tumor growth, invasion and metastasis. However, little is known about its hematopoietic activity during malignant development and progression. Here we show that in a mouse tumor model, tumor-derived VEGF acts as an endocrine-like hormone to induce extramedullary hematopoiesis by targeting distal organs in the host. In tumor-bearing mice, circulating VEGF induced hepatomegaly and splenomegaly owing to vessel dilation, tortuosity and activation of hematopoiesis. Furthermore, VEGFR1 and VEGFR2 were primarily localized in blood vessels rather than hepatocytes or splenocytes, demonstrating that alteration of angiogenic profiles modulates hematopoiesis in these organs. Stimulation of extramedullary hematopoiesis sheds new light on complex biological functions of VEGF and significantly increases our understanding of molecular mechanisms underlying VEGF-induced tumor growth.

## Introduction

Malignant tissues produce multiple growth factors and cytokines to induce angiogenesis, which is essential for tumor growth, invasion and metastasis [1,2]. Among tumor-derived angiogenic factors, vascular endothelial growth factor (VEGF) is probably one of the best-characterized molecules. VEGF displays multiple physiological and pathological functions by targeting both vascular and non-vascular systems [3-5]. In developing embryos, deletion of only one allele of the *vegf *gene results in severe defects of early embryos that manifest impaired hematopoiesis and collapse of the vascular system [6,7]. In the adult, VEGF is required to maintain the integrity of the vasculature and physiological functions, including endothelial survival, vascular fenestration in several endocrine glands, neurotrophic effects, support of bone marrow hematopoiesis, wound healing, and reproductive activity [8,9]. To maintain these multiple physiological functions, optimal levels of VEGF expression are required in various tissues and organs [10,11]. When optimal expression levels are altered, VEGF often causes pathological disorders by triggering uncontrolled vascular responses that include pathological vasculogenesis, angiogenesis, and tissue edema. The plasticity features of VEGF expression determine its involvement in a broad spectrum of human diseases including malignant and non-malignant disorders. For example, tissue hypoxia is one of the key factors that elevate VEGF expressions in both tumors and non-malignant disorders [12,13].

In tumors, VEGF is known to significantly contribute to pathological angiogenesis, tortuosity of tumor vasculatures and vasculogenesis, which all together lead to accelerated growth rates of tumors, invasion and metastasis [14]. In addition to vascular effects, VEGF also mobilizes mononucleic cells and probably endothelial progenitor cells from bone marrow, whereas former enhances tumor inflammation, the latter participates in vasculogenesis [15,16]. VEGF-induced vascular tortuosity and leakiness also provide a structural basis for tumor cell invasion into the circulation system, leading to distal metastasis. In addition to hematologous metastasis, recent work from our laboratory and others demonstrates that VEGF is also a potent lymphangiogenic factor, which promotes lymphatic metastasis [17-20].

Similar to the prototype member of VEGF, other members in the VEGF family exhibit overlapping and sometimes distinctive biological functions in both physiological and pathological settings, depending on their abilities to activate a subset of VEGF receptors. While VEGFR2 is consistently reported as a functional receptor to transduce angiogenic, vasculogenic and vascular permeability activities, functional properties of VEGFR1 remain controversial [21]. Probably, VEGFR-1 transduces both positive and negative signals in endothelial and non-endothelial cells, depending on the choice of experimental settings [21]. Additionally, high molecular forms of VEGF also bind to neuropilin-1 and -2, which are involved in tumor growth and metastasis [22,23].

Owing to the pivotal role of VEGF in regulation of pathological angiogenesis and tumor growth, several anti-VEGF drugs have been developed for cancer therapy. These include neutralization of the VEGF ligand by antibodies such as bevacizumab and small chemical compounds targeting the receptor signaling pathways such as sorafenib and sunitinib [24]. In general, clinical responses to these drugs in combinations with chemotherpy are very encouraging and they become one of the key components of the first-line therapeutic regimens for various human cancers [25]. In the present study, we provide new evidence that VEGF could induce extramedullary hemetapoiesis in adult tumor-bearing mice. These findings suggest that VEGF may significantly contribute to tumor growth via improvement of hematopoiesis

## Methods

### Animals and reagents

Female C57Bl/6 mice were anesthetized by Isoflurane (Abbott Scandinavia) before all procedures. The experiments were followed up to 4 weeks. Mice were sacrificed by exposure to a lethal dose of CO_2 _followed by cervical dislocation. All animal studies were reviewed and approved by the animal care committee of the North Stockholm Animal Board (Stockholm, Sweden). Antibodies include a rat anti-mouse CD31 monoclonal antibody, a rat anti-mouse erythroid cells (Ter119) monoclonal antibody (BD Pharmingen, San Diego, CA, USA), a rat anti-mouse VEGFR1 (MF1) antibody, a rat anti-mouse VEGFR2 (DC101) antibody (ImClone Systems Inc., NY, USA), and a rabbit anti-mouse polyclonal VEGFR-2 antibody (kindly provided by Dr. Rolf Brekken at University of Texas Southwestern Medical Center).

### Cell culture

Murine T241 fibrosarcoma is transfected with control vector and DNA constructs which stably express human VEGF_165 _as previously described [5,26]. Tumor cell lines were grown and maintained in Dulbecco's Modified Eagles Medium (DMEM) (Sigma) with 10% heat-inactivated fetal bovine serum.

### Xenograft tumor model

Approximately 1 × 10^6 ^tumor cells were subcutaneously injected into the dorsal region of each mouse. Tumor growth was monitored on a daily basis. When tumors reached approximately 1.0 cm^3^, the experiments were terminated. Various tissues and organs were dissected and fixed with 4% paraformaldehyde (PFA) overnight, followed by transferring into PBS until further analysis. For some analyses, a portion of each tissue was frozen at -80°C until further use. In blockade experiments, VEGF tumor-bearing mice were randomly divided into two groups (n = 6-8/group) and received VEGFR1 or VEGFR2 blockades as previous described [4,5]. The treatment started at day 2 after tumor implantation. Each mouse in various groups received vehicle, anti-VEGFR1 (MF1, Imclone Inc., 800 μg/mouse) or anti-VEGFR2 (DC101, Imclone Inc., 800 μg/mouse) antibodies twice a week for a total 2-week period.

### Histological analysis and immunohistochemistry

Paraffin-embedded tissues including liver, spleen and bone were sectioned in 5 μm thickness and stained with hemtoxylin/eosin (H&E) according to a standard method [5]. Liver sections were stained with an anti-Ter119 antibody as the primary antibody. After extensive washes with PBS, sections were stained with a secondary antibody of HRP-conjugated anti-rat IgG. Positive signals were developed by substrate DAB, followed by capturing under a light microscope (40× magnification).

Cryostat tissue sections of 10 μm thickness were stained according to a standard immunohistochemical procedure [5]. Briefly, sections were incubated with primary antibodies, including anti-CD31, Ter119, VEGFR1 or VEGFR2, overnight at 4°C. After rigorous washes with PBS for three times, sections were incubated for 1 hour at room temperature with various secondary antibodies, including a goat anti-rat Alexa-555-labeled antibody (1:500), a FITC-labeled rabbit anti-rat IgG (1:300, Vector Laboratories), a Cy5-labeled goat anti-rabbit antibody (1:400, Chemicon International, Temecula, CA, USA), which were used for either mono- or double-staining. Sections were mounted on glass-slides with Vectashield mounting medium (Vector Laboratories). Positive signals were photographed under a confocal microscopy (20×, Zeiss Confocal LSM510 Microscope).

## Results and Discussion

**Figure 1:**
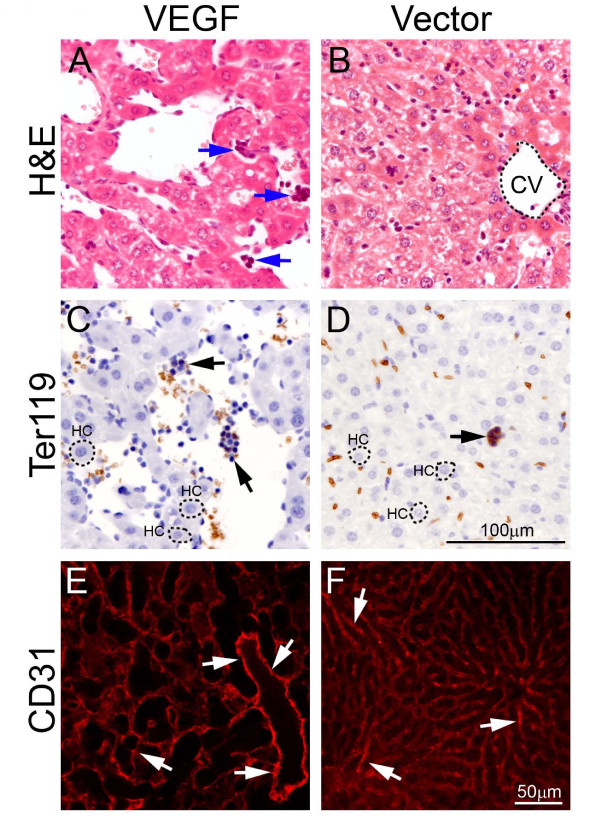
**Hepatic hematopoietic islets and vasculature**. Liver sections from T241-VEGF and T241-vector tumor-bearing mice were stained with H&E (A and B), an anti-Ter119 antibody (C and D), or anti-CD31 antibody (E and F). Arrows in panels A, C, and D point to hematopoietic islets. Arrows in E and F point to hepatic sinusoidal microvessels. CV = central vein. HC = hepatocyte. Bar in panels A-D = 100 μm. Bar in panels E and F = 50 μm.

Our recent findings show that tumor-derived VEGF induces a systemic syndrome in mice, manifesting a cancer-associated paraneoplastic syndrome [5]. Using the same tumor model, ie., murine T241 fibrosarcoma model, we studied the role of VEGF in modulation of hematopoiesis. In a xenograft model, implantation of T241-VEGF tumors in mice led to hepatomegaly and splenomegaly [5]. Histological examination of liver tissues showed that visible hematopoietic islets in liver sections from T241-VEGF tumor-bearing mice but not in liver sections of T241-vector control tumor-bearing mice (Fig. 1). To further validate the identity of hematopoietic islets, liver sections were stained with Ter119, a hematopoietic marker for erythroblasts. Consistent with H&E staining, these morphologically hematopoietic islets exhibited positive signals of Ter119 (Fig. 1). Quantification analysis showed a significant difference between T241-VEGF and T241-vector groups (see ref [5]). Moreover, the sinusoidal hepatic vasculature became highly dilated in livers of T241-VEGF tumor-bearing mice but not in livers of T241-vector tumor-bearing mice (Fig. 1). These findings demonstrate that tumor-derived VEGF significantly contributes hepatic hematopoiesis and the vascular architecture is significantly altered in this organ.

**Figure 2:**
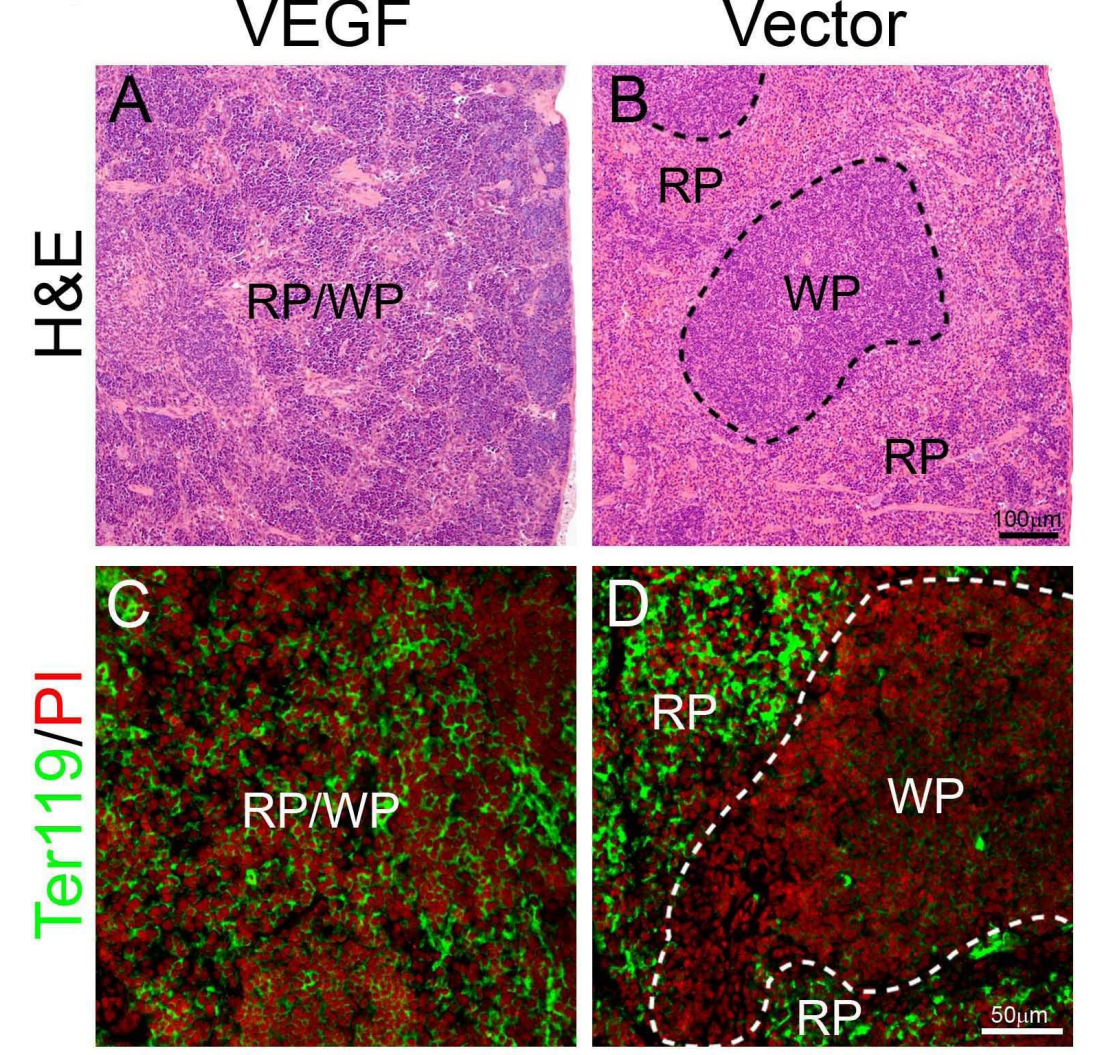
**Spleno-extramedullary hematopoiesis**. Spleen sections from T241-VEGF and T241-vector tumor-bearing mice were stained with H&E (A and B), or were double stained an anti-Ter119 antibody and propidium iodide (PI) (C and D). Dashed lines in panel B encircle white pulp (WP). RP = red pulp. Hematopietic cells from the erythrocyte lineage were labeled with Ter119 (green) and all cell nuclei were labeled with PI (red). Bar in panel A and B = 100 μm. Bar in C and D = 50 μm.

Because extramedullary hematopoiesis often occurs in the spleen of mice, we next examined spleens of tumor-bearing mice. Similar to livers, splenomegaly also existed in T241-VEGF tumor-bearing mice [5]. Histological examination showed that apparent borders between the white pulp (WP) and red pulp (RP) under physiological conditions were vanished and were replaced by a mixture of WP and RP without any distinctive borders throughout the entire spleen of T241-VEGF tumor-bearing mice (Fig. 2). Ter119 staining revealed that active hematopoiesis occurred throughout the entire spleen tissue of T241-VEGF tumor-bearing mice (Fig. 2). In markedly contrast, Ter119 positive signals only present in the RP region in the spleen of T241-vector tumor-bearing mice (Fig. 2). These findings show that tumor-derived VEGF significantly modulates extramedullary hematopoiesis in the adult spleen by expansion of RP areas.

**Figure 3:**
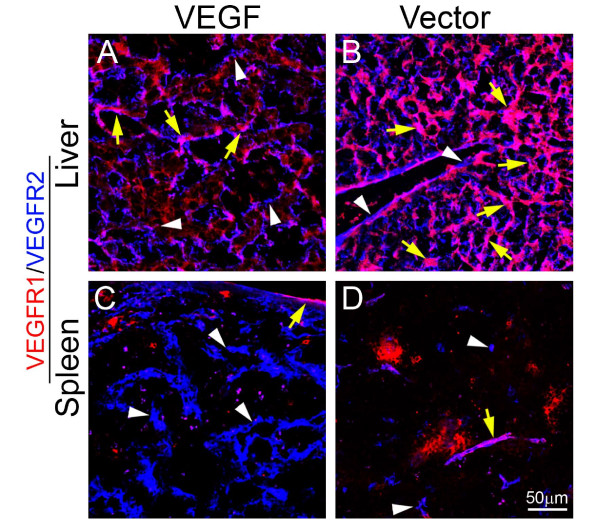
**Localization of VEGFR1 and VEGFR2 in liver and spleen tissue sections**. Liver (A and B) and spleen (C and D) tissue sections from T241-VEGF and T241-vector tumor-bearing mice were double stained with monoclonal antibodies specific for mouse VEGFR1 (red) or VEGFR2 (blue). Yellow arrows point to double positive signals and white arrowheads indicate single positive singles. Bar = 50 μm.

**Figure 4:**
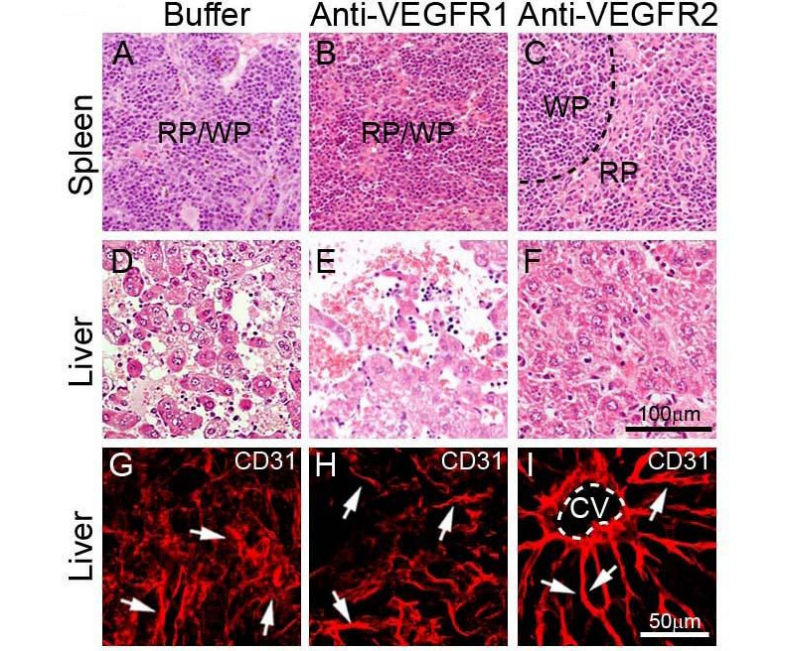
**Histology and vasculature of liver and spleen treated with VEGFR blockades**. Spleen (A-C) and liver (D-F) tissue sections from buffer- (A, D, and G), anti-VEGFR1- (B, E and H), or anti-VEGFR2-(C, F and I) treated T241-VEGF-bearing mice were stained with H&E (A-F) or anti-CD31 (G-I). Arrows in panels of G-I indicate microsinusoidal vasculatures in liver sections. CV = central vein. Bar = 50 μm.

To delineate molecular mechanisms underlying systemic VEGF-induced extramedullary hematopoiesis, VEGFR1 and VEGFR2 were detected in both liver and spleen tissues. Interestingly, expression patterns of VEGFR1 and VEGFR2 were restricted in blood vessels, but not in other cell types including hepatocytes, splenocytes and stromal cells (Fig. 3). Moreover, VEGF2 positive signals were generally enhanced while VEGFR1 signals were decreased in both spleens and livers of T241-VEGF tumor-bearing mice (Fig. 3). To further distinguish VEGFR1- and VEGFR2-mediated signaling pathways in hematopoiesis, VEGFR1 and VEGFR2 specific blockades were used for treatments. An anti-VEGFR2 specific monoclonal antibody completely restored histological structures of liver and spleen in VEGF tumor-bearing mice, whereas VEGFR1 blockade had no effect in these organs (Fig. 4). Consistent with histological changes, VEGFR2 blockade completely normalized hepatic sinusoidal vasculatures whereas VEGFR1 blockade lacked such an effect. These findings demonstrate that VEGFR2 is a crucial receptor that mediates extramedullary hematopiesis and tortuosity of vasculatures in these organs.

**Figure 5:**
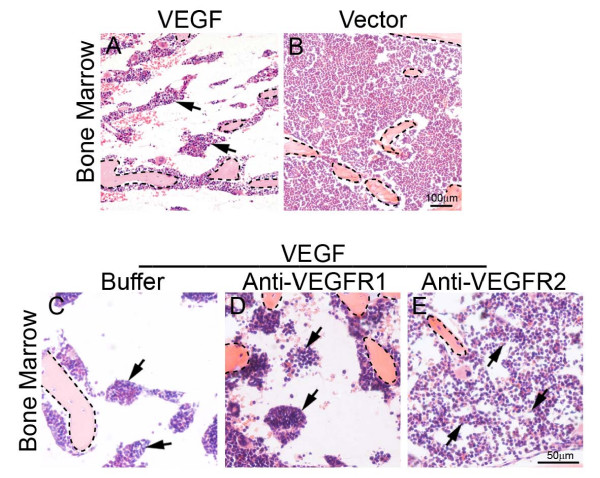
**Histological examination of bone marrow**. Bone marrows of T241-VEGF (A) and T241-vector (B) tumor-bearing mice were stained with H&E. Bone marrows of buffer- (C), anti-VEGFR1- (D), or anti-VEGFR2-(E) treated T241-VEGF-bearing mice were stained with H&E. Arrows in panels A and C-E point to the residual hematopoietic islets. Bone matrix were encircled with dashed lines. Bar in panel A and B = 100 μm. Bar in panels C-E = 50 μm.

Although VEGF might directly promote extramedullary hematopoiesis in liver and spleen, it was plausible that VEGF-induced extramedullary hematopoiesis echoes defective bone marrow (BM) hematopoiesis and thus represents a compensatory mechanism of BM deficiency. To test this possibility, we histologically examined BM of VEGF tumor-bearing mice. Markedly, VEGF tumor-bearing mice suffered from a severe defect in BM by loosing hematopoiestic cells relative to vector control BM (Fig. 5). Interestingly, VEGFR2 blockade could largely restore VEGF-induced BM defects while VEGFR1 blockade was unable to rescue the defective phenotype (Fig. 5). Based on these findings, it is likely that extramedullary hematopoiesis resulted from defective BM hematopoiesis via compensatory activation of this process in liver and spleen.

The data presented in this study provide compelling evidence that tumor-derived VEGF displays a profound effect on the hematopoietic system. It appears that tumor-derived VEGF enters into the circulation and acts on either endothelial cells and/or hematopietic progenitor cells to modulate hematopoiesis. Although molecular mechanisms underlying VEGF-induced impairment of BM hematopoiesis remain unknown, it is possible that VEGF mobilizes BM hematopoietic stem cells to accumulate in peripheral tissues and organs. Indeed, VEGF has been reported to mobilize BM cells in tumor models and other experimental settings [15,16]. Suppression of BM hematopoiesis would in theory result in a decreased tumor growth rate due to insufficient oxygen supply. In contrast, in our experimental system we have found that VEGF tumors grow at an accelerated rate relative to control tumors [5,26]. There are several possible mechanisms to explain these paradoxical findings: 1) VEGF also exhibits potent angiogenic and vasculogenic activities, which significantly facilitate blood perfusion in tumor tissues; 2) Hypoxia is known to increase tumor invasion and spread. Indeed, necrotic cores are often observed in VEGF tumors relative to control tumors. These findings suggest that VEGF tumors suffer from higher degrees of tissue hypoxia than control tumors, and hypoxia would persuade tumor cells to expand to healthy neighboring tissues; 3) VEGF-mobilized BM-derived inflammatory cells significantly contribute to tumor growth by producing a range of angiogenic factors and cytokines, and 4) Extramedullary hematopoiesis compensates oxygen supply and supports tumor growth. However, this compensatory mechanism may not sufficiently rescue the defective phenotype of BM hematopoiesis.

Our findings also show that VEGF targeted therapy has a profound impact on multiple tissues and organs [5]. In the hematopoietic system, anti-VEGF drugs might significantly improve BM hematopoiesis and anemia in patients. However, the distinct functions mediated by VEGFR1 and VEGFR2 should be separated. While VEGFR1 is distributed in hematopoietic cells such as monocytes and neutrophils, VEGFR2 is the key receptor to mediate hematopoietic signals [5]. Consistent with our findings, genetic deletion of vegfr2 gene in mice results in severe defects of both vascular and hematopoietic systems in mouse embryos [6,7]. In contrast, deletion of VEGFR1 does not seem to significantly affect hematopoiesis [6,7]. Most currently available drugs such as bevacizumab, sunitinib and sorafenib do not allow us to distinguish the biological functions mediated by VEGFR1 and VEGFR2 [21]. It is unclear if VEGFR2 specific blockade would produce better clinical outcomes than currently available VEGF inhibitors in the clinic. However, it is known that VEGFR1 could also mediate negative signals of angiogenesis under both physiological and pathological conditions [21].

Neutralization of VEGF-induced systemic disorders in cancer patients is probably an important mechanism for cancer therapy although antiangiogenesis is one of the key mechanisms of anti-VEGF drugs. Consistent with this notion, suppression of tumor size has not been positively correlated with clinical benefits of these antiangiogenic drugs [27]. Our current findings provide new evidence that VEGF targets multiple tissues and organs and that anti-VEGF therapy may have a global impact in cancer patients.

## Conclusions

Our findings show that tumor-derived VEGF modulates the hematopoietic system by suppression of BM-hematopoiesis, leading to activation of extramedullary hematopoiesis in liver and spleen. Modulation of hematopoiesis is mediated by VEGFR2 but not by VEGFR1. These results demonstrate that tumor-derived VEGF has a profound impact on multiple tissues and organs and these broad systemic effects might further facilitate tumor growth and metastasis. Additionally, circulating VEGF derived from the malignant tissue also contributes to development of cancer-associated systemic disorders, which significantly jeopardize quality of life and survival of cancer patients. Thus, anti-VEGF therapy may produce more profound beneficial effects in cancer patients by improving hematopoiesis and other systemic functions of cancer patients.

## List of abbreviations

VEGF: vascular endothelial growth factor; VEGFR: VEGF receptor; BM: bone marrow; LLC: Lewis lung carcinoma; RP: red pulp; WP: white pulp.

## Competing interests

The authors declare that they have no competing interests.

## Authors' contributions

YX and YC designed research; YX, FC, SL and DZ performed research; YX, FC, SL, DZ and YC analyzed data; YC wrote the paper.
